# Tract-Based Spatial Statistics Analysis of Diffusion Tensor Imaging in Older Adults After the PICMOR Intervention Program: A Pilot Study

**DOI:** 10.3389/fnagi.2022.867417

**Published:** 2022-06-03

**Authors:** Hikaru Sugimoto, Mihoko Otake-Matsuura

**Affiliations:** RIKEN Center for Advanced Intelligence Project, Tokyo, Japan

**Keywords:** cognitive intervention, conversation, diffusion tensor imaging, PICMOR, tract-based spatial statistics

## Abstract

Diffusion tensor imaging (DTI) enables the investigation of white matter properties *in vivo* by applying a tensor model to the diffusion of water molecules in the brain. Using DTI metrics including fractional anisotropy (FA), mean diffusivity (MD), axial diffusivity (AD), and radial diffusivity (RD), an attempt has been made to detect age-related alterations in the white matter microstructure in aging research. However, the use of comprehensive DTI measures to examine the effects of cognitive intervention/training on white matter fiber health in older adults remains limited. Recently, we developed a cognitive intervention program called Photo-Integrated Conversation Moderated by Robots (PICMOR), which utilizes one of the most intellectual activities of daily life, conversations. To examine the effects of PICMOR on cognitive function in older adults, we conducted a randomized controlled trial and found that verbal fluency task scores were improved by this intervention. Based on these behavioral findings, we collected in this pilot study diffusion-weighted images from the participants to identify candidate structures for white matter microstructural changes induced by this intervention. The results from tract-based spatial statistics analyses showed that the intervention group, who participated in PICMOR-based conversations, had significantly higher FA values or lower MD, AD, or RD values across various fiber tracts, including the left anterior corona radiata, external capsule, and anterior limb of the internal capsule, compared to the control group, who participated in unstructured free conversations. Furthermore, a larger improvement in verbal fluency task scores throughout the intervention was associated with smaller AD values in clusters, including the left side of these frontal regions. The present findings suggest that left frontal white matter structures are candidates for the neural underpinnings responsible for the enhancement of verbal fluency. Although our findings are limited by the lack of comparable data at baseline, we successfully confirmed the hypothesized pattern of group differences in DTI indices after the intervention, which fits well with the results of other cognitive intervention studies. To confirm whether this pattern reflects intervention-induced white matter alterations, longitudinal data acquisition is needed in future research.

## Introduction

Diffusion tensor imaging (DTI) is a neuroimaging technique that enables researchers to investigate white matter properties *in vivo* by applying a tensor model to the diffusion of water molecules in the brain (Rowe et al., 2016). In aging research, an attempt has been made to detect age-related structural changes in white matter fibers using DTI indices, including fractional anisotropy (FA), mean diffusivity (MD), axial diffusivity (AD), and radial diffusivity (RD; Madden et al., 2009, 2012; Bennett and Madden, 2014). However, a relatively small number of studies have used comprehensive DTI metrics to assess the effects of cognitive intervention/training on white matter fiber health in older adults (Wassenaar et al., 2019). Using DTI measures comprehensively, this pilot study aimed to characterize white matter fiber tracts in older adults who participated in a conversation-based intervention program named Photo-Integrated Conversation Moderated by Robots (PICMOR; Otake-Matsuura et al., 2021).

Normal aging is associated with changes in white matter microstructure (Madden et al., 2009, 2012; Bennett and Madden, 2014). For example, a large-scale DTI study using the United Kingdom Biobank resource reported that older age was associated with decreased FA and increased MD, AD, and RD across numerous white matter tracts, including association fibers, such as the inferior fronto-occipital, inferior longitudinal, superior longitudinal, and uncinate fasciculi, and thalamic radiation fibers, such as the anterior, superior, and posterior thalamic radiation, as well as the forceps minor (Cox et al., 2016). To prevent or slow age-related changes in cognitive function and white matter, various cognitive intervention/training paradigms have been developed (Wassenaar et al., 2019). Previous studies have attempted to elucidate intervention/training-induced white matter changes using DTI (Lovden et al., 2012; Chapman et al., 2015; Nozawa et al., 2015; Antonenko et al., 2016; Fissler et al., 2017; Dziemian et al., 2021). However, the use of comprehensive DTI metrics to assess intervention/training effects on white matter fiber health in older adults remains limited (Lovden et al., 2010; Engvig et al., 2012; Strenziok et al., 2014; Lampit et al., 2015; Cao et al., 2016; de Lange et al., 2016, 2017; Youn et al., 2019). One such study reported that older adults who were assigned to the training group and received memory strategy training for 10 weeks showed a less age-related decline in white matter microstructure, including the corpus callosum, corticospinal tract, cingulum bundle, superior longitudinal fasciculus, and anterior thalamic radiation, than those assigned to the control group (de Lange et al., 2017). From baseline to follow-up, the control group showed a greater decrease in FA and a greater increase in MD, AD, and RD relative to the training group in these fiber tracts. Another study reported that the intervention group who practiced multi-domain cognitive training tasks, including working memory, episodic memory, and perceptual speed tasks, in about 100 daily sessions over approximately 6 months increased FA values and decreased MD and RD values in the genu of the corpus callosum between the pre- and post-intervention period, while no significant change in these metrics was identified in the control group (Lovden et al., 2010). Taken together, although the fibers considered to reflect intervention/training effects differ among studies, possibly due to differences in intervention/training methodologies, a consistent pattern can be seen in the DTI indices of the post-intervention period: larger FA values or smaller values in other metrics in the intervention group compared to the control group.

In terms of availability and sustainability, it is important to design a cognitive intervention paradigm that utilizes daily life activities. Social activity is one of the most intellectual activities of daily life and the level of activity engagement is related to cognitive function and white matter microstructure in older adults (Kelly et al., 2017; Anaturk et al., 2018); hence, it can be incorporated into such intervention paradigms. Of the various forms of social activity, conversation is a promising approach to improving or maintaining cognitive health in older adults because it requires elaborate cognitive processes, such as organizing one’s thoughts and understanding others’ ideas (Dodge et al., 2015). The PICMOR program, which we recently developed, is a cognitive intervention method based on group conversations (Otake-Matsuura et al., 2021). In this method, conversations among group members are prompted and chaired by a robot. The robotic management enables equal allocation of speaking time to everyone, and each participant is encouraged by the robot to talk about a topic within the allocated time. The system also enables giving everyone equal discussion time, and the participants are required to ask and answer questions. In the discussion period, the robot monitors the utterances of each participant in real-time and automatically encourages and stops their utterances to guarantee equal amounts of speaking time. The nature of this communication task involves exercising executive functions, such as flexibility, planning, working memory, and response inhibition, by encouraging the participants to talk within a limited time, to have a flexible discussion by asking and answering questions, to temporarily store and manipulate information necessary to ask questions, and to refrain from interrupting other group members. Thus, we expected that cognitive ability involving executive functions, such as the ability to produce words within a certain length of time, would be better trained by group conversations employing the PICMOR method than by conventional group conversations.

Recently, we conducted a randomized controlled trial (RCT) to assess the effects of interventions involving this method on cognitive function in older adults (Otake-Matsuura et al., 2021). Consistent with our idea, the results of the phonological verbal fluency task, in which the ability to produce as many words as possible starting with a designated letter within a limited time is tested (Fujiwara et al., 2010; Lezak et al., 2012), showed a significantly greater improvement in the intervention group, who participated in the PICMOR program, than in the control group, who participated in unstructured free conversations among group members without robotic assistance. Based on the behavioral results, we assumed that differences in white matter properties underlie group differences in cognitive enhancement. Specifically, we hypothesized that if such difference was measurable, it would emerge as higher FA values or lower MD, AD, or RD values in the intervention group compared to the control group. Such a pattern in DTI metrics was based on previous findings from cognitive intervention studies that used comprehensive DTI indices to assess the intervention/training effects on white matter fiber health in older adults (Lovden et al., 2010; Engvig et al., 2012; Cao et al., 2016; de Lange et al., 2017). Given the evidence from neuropsychological studies demonstrating the contribution of the left frontal region to verbal fluency (Stuss et al., 1998; Baldo et al., 2006, 2010; Robinson et al., 2012; Biesbroek et al., 2016, 2021; Chouiter et al., 2016; Li et al., 2017; Thye et al., 2021), white matter structures in this region may show the assumed DTI pattern. This idea is also supported by our preliminary findings from a resting-state functional magnetic resonance imaging (MRI) study (Sugimoto et al., 2020) showing that the left cortical frontal area, which is a core region for verbal fluency (Costafreda et al., 2006; Wagner et al., 2014), had differential functional connectivity between the two groups. The purpose of this pilot study was to identify candidate structures for white matter alterations induced by conversation-based interventions, which were also associated with enhanced verbal fluency, by examining the hypothesized pattern in DTI measures after the intervention period.

## Methods

### Participants

All participants of our previous RCT (Otake-Matsuura et al., 2021) were recruited, and 61 out of 65 participants (31 and 30 in the intervention and control groups, respectively) participated in this additional MRI experiment. As previously reported (Sugimoto et al., 2020; Sugimoto and Otake-Matsuura, 2022), no significant group differences were found in terms of age [the intervention group, mean ± standard deviation (*SD*) = 72.84 ± 3.45 years; the control group, mean ± *SD* = 72.03 ± 2.72 years], sex (the intervention group, 15 females and 16 males; the control group, 17 females and 13 males), and educational level (the intervention group, 20 people with education for 13 years and more; the control group, 17 people with education for 13 years and more). The MRI data from four RCT participants were not available for the following reasons: claustrophobia, being equipped with a pacemaker, or declining to participate in the MRI scans. All participants provided written informed consent for the protocol, which was approved by the Institutional Review Board of RIKEN. The participants were right-handed and native Japanese-speaking individuals. The demographic and behavioral data from pre/post cognitive tests, including the verbal fluency task, are detailed elsewhere (Sugimoto et al., 2020; Sugimoto and Otake-Matsuura, 2022).

### Intervention Procedures

Details of the intervention procedures have been described in our previous study (UMIN000036667; Otake-Matsuura et al., 2021). Briefly, 72 community-dwelling older adults were recruited from the Silver Human Resources Center for our previous RCT. Based on screenings and baseline assessments, we excluded participants meeting the following criteria: dementia, neurological impairment, any disease or medication known to affect the central nervous system, and scoring less than 24 in the Japanese version of the Mini-Mental State Examination (MMSE-J; Sugishita et al., 2018). Consequently, 65 people were enrolled and randomly assigned to the intervention or control groups. The intervention period lasted for 12 weeks, during which both the intervention and control groups participated in group conversations once a week. The intervention period was followed by a post-assessment of cognitive function. Finally, the MRI experiment was conducted.

For group conversations during the intervention period, both the intervention and control groups were divided into eight subgroups, each with four members (except for one control subgroup with five members), and instructed to talk with other members of the subgroup. In the group conversation provided for the control group, participants joined unstructured free conversations where they talked freely among subgroup members, as they would converse in daily life. By contrast, the group conversation provided for the intervention group was controlled by a robotic assistive system, in which a robot acted as a chairperson and assisted the conversation by encouraging each participant to describe their daily life experiences and discuss them with other group members. Each participant was prompted by the robot to talk about an event along a predetermined theme for 1 min using a photo displayed on the screen they had taken beforehand. The 1-min talking period was repeated to explain another event related to the same theme using another photo. During this period, the other members of the subgroup were required to listen carefully and ask questions later. Following this, a 2-min discussion period was provided, during which the participant had to answer questions raised by other group members. The 2-min discussion period was repeated to discuss the second event. During the discussion period, the robot monitored the utterances of each member in real-time and controlled the conversations by encouraging or stopping utterances to balance the amount of talking time for each person. Using this robotic assistive system, strict time management and automatic turn-taking based on the actual speech time of each participant were achieved. All members were provided with 1-min talking periods and 2-min discussion periods.

### Data Acquisition and Analysis

All MRI data were collected after the intervention period using a Philips Achieva 3.0 Tesla scanner at the Advanced Imaging Center Yaesu Clinic, Tokyo. During the scanning, the participants’ head movements were minimized by a belt and foam pads, and the participants were required to stop their body motion. Diffusion-weighted images were acquired using a single-shot, spin-echo, echo-planar imaging pulse sequence with the following parameters: repetition time = 6,968 ms, echo time = 69 ms, field of view = 35.0 × 35.0 cm, matrix size = 128 × 128, slice thickness/gap = 2.7/0 mm, 59 horizontal slices, 40 diffusion gradient directions with *b*-value = 1,000 s/mm^2^, and an additional volume with *b-*value = 0 s/mm^2^. The data were visually inspected for imaging artifacts. In addition, T1-weighted structural and resting-state functional images were obtained. The results of these anatomical and functional scans have been reported previously (Sugimoto et al., 2020; Sugimoto and Otake-Matsuura, 2022).

Diffusion-weighted images were analyzed using Functional MRI of the Brain (FMRIB)’s Software Library (FSL) software version 6.0.3^1^ (Smith et al., 2004; Jenkinson et al., 2012) implemented in Lin4Neuro 18.04^2^ (Nemoto et al., 2011). The images were preprocessed using tools and scripts provided by MRtrix3^3^ (Tournier et al., 2019) in accordance with the following procedure. First, the *dwidenoise* command was executed to reduce thermal noise (Veraart et al., 2016a, b). Second, the *mrdegibbs* command was used to reduce Gibbs-ringing artifacts (Kellner et al., 2016). Third, motion and eddy-current distortions were corrected using the *dwipreproc* command, in which the *-rpe_none* option was chosen because inverted phase-encoding image data, i.e., anterior-to-posterior, were not available (Andersson and Sotiropoulos, 2016). In addition, the *–slm=linear* option was employed in this step. Fourth, B1 field inhomogeneity correction was performed using the *dwibiascorrect* command with the *-ants* option (Tustison et al., 2010). Finally, the *dwi2mask* command was executed to generate a whole-brain mask from the preprocessed dataset. Using the preprocessed data and whole-brain mask, a diffusion tensor model was fitted at each voxel using FSL’s *dtifit* program, a part of the FMRIB’s Diffusion Toolbox (FDT). The default outputs of this procedure included whole-brain maps of AD, MD, and FA computed from the first, second, and third eigenvalues (λ_1_, λ_2_, and λ_3_, respectively) corresponding to the three directions of water diffusivity, as follows: The RD maps were created using the *fslmaths* command.


$$AD=\lambda_{1}$$

$$RD=\frac{\lambda_{2}+\lambda_{3}}{2}$$

$$MD=\frac{\lambda_{1}+\lambda_{2}+\lambda_{3}}{3}$$

$$FA=\sqrt{\frac{{\left(\lambda_{1}-\lambda_{2}\right)}^{2}+{\left(\lambda_{2}-\lambda_{3}\right)}^{2}+{\left(\lambda_{3}-\lambda_{1}\right)}^{2}}{2\left(\lambda_{1}^{2}+\lambda_{2}^{2}+\lambda_{3}^{2}\right)}}$$

Voxel-based statistical analysis of the DTI metrics was performed using FSL’s tract-based spatial statistics (TBSS) as follows (Smith et al., 2006). First, the FA images were preprocessed using the *tbss_1_preproc* command to eliminate probable outliers from the diffusion tensor fitting. Second, the *tbss_2_reg -T* command was executed to perform nonlinear registration, aligning the FA images of all participants into the FMRIB58_FA_1mm standard-space image. Third, using the *tbss_3_postreg -S* command, each participant’s FA image was affine-transformed into the Montreal Neurological Institute (MNI152) standard space. In this step, all individual FA images were averaged to create a mean FA image, and this image was applied to generate a mean FA skeleton that represents the center of the fiber tracts common to all participants. For the mean FA skeleton, a threshold was set at 0.2 with the *tbss_4_prestats* command. Executing this command resulted in projecting all individual FA images onto the mean FA skeleton and generating a four-dimensional image file containing the skeletonized FA data for all participants. Finally, using a binary mask of the FA skeletonized image as a mask image, a voxel-wise statistical comparison between the intervention and control groups was performed on the four-dimensional skeleton image file using FSL’s *randomise* program (Winkler et al., 2014) with the number of random permutations set to 5,000 (Nichols and Holmes, 2002). Data for the other DTI metrics, including MD, AD, and RD, were analyzed using the *tbss_non_FA* script to generate a four-dimensional skeleton image file for each of the other DTI metrics. These files were then used for statistical comparisons using the *randomise* program (Winkler et al., 2014). All metrics were analyzed using the general linear model built using the FEAT toolbox with a higher-level design. The statistical model included age, sex, educational level, and total intracranial volume (TIV; Takao et al., 2011, 2014) as covariates of no interest. TIV was estimated in our previous voxel-based morphometry study (Sugimoto and Otake-Matsuura, 2022). As a complementary analysis, we also conducted a regression analysis for each DTI index using the *randomise* program (Winkler et al., 2014) with the increase in verbal fluency task scores from pre-intervention to post-intervention (i.e., post-minus pre-intervention) as the regressor. This model also included age, sex, educational level, and TIV as nuisance covariates. In all analyses, the statistical threshold was set at *p* < 0.05, corrected for multiple comparisons by controlling the family-wise error rate and employing the threshold-free cluster enhancement method (Smith and Nichols, 2009). The anatomical sites of significant clusters were primarily defined using the Johns Hopkins University (JHU) International Consortium of Brain Mapping (ICBM)-DTI-81 white matter labels atlas (Mori et al., 2008) implemented in the FSL.

## Results

**Figure 1:**
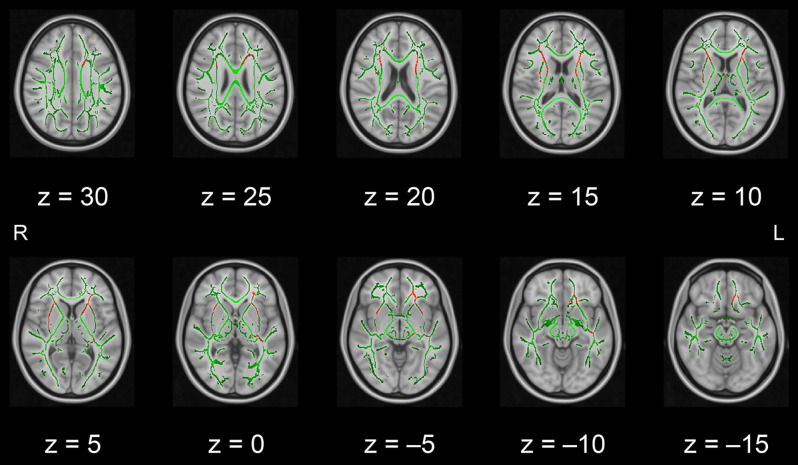
White matter fiber tracts showing significantly higher FA values in the intervention group than in the control group (displayed in red). The results are overlaid on the mean FA skeleton (shown in green) and the standard MNI152 T1 1-mm^3^ brain template. The value of *z* in the horizontal plane represents the MNI *z*-coordinate. Abbreviation: FA, fractional anisotropy; L, left; MNI, Montreal Neurological Institute; R, right.

**Figure 2:**
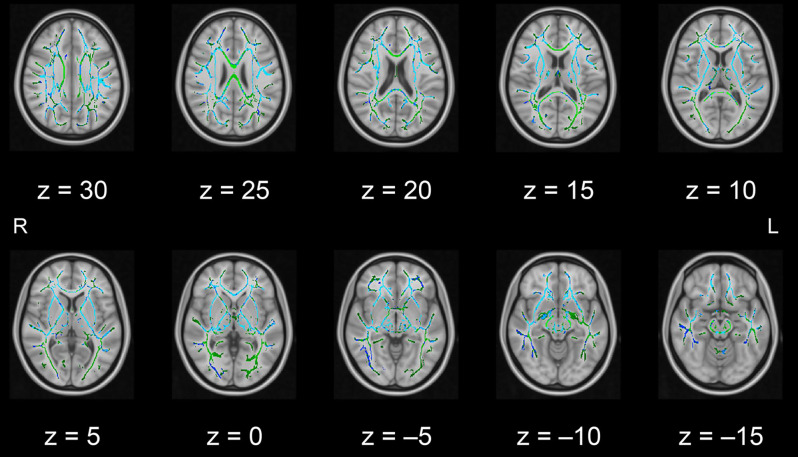
White matter fiber tracts showing significantly lower MD values in the intervention group than in the control group (displayed in blue). The results are overlaid on the mean FA skeleton (shown in green) and the standard MNI152 T1 1-mm^3^ brain template. The value of *z* in the horizontal plane represents the MNI *z*-coordinate. Abbreviation: FA, fractional anisotropy; L, left; MD, mean diffusivity; MNI, Montreal Neurological Institute; R, right.

**Figure 3:**
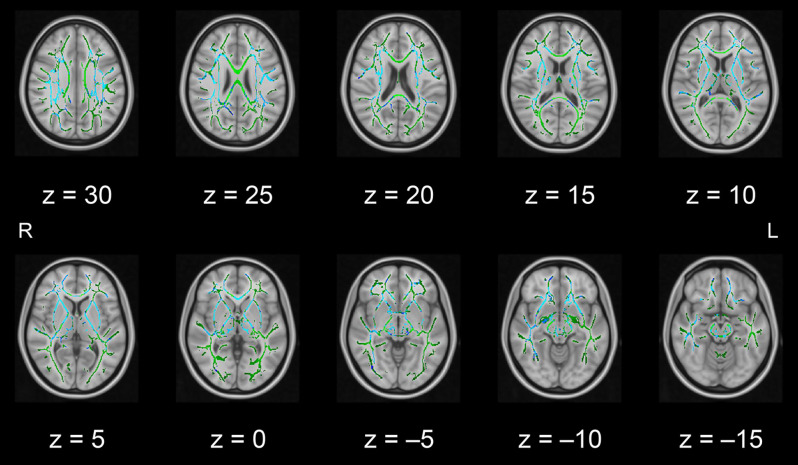
White matter fiber tracts showing significantly lower AD values in the intervention group than in the control group (displayed in blue). The results are overlaid on the mean FA skeleton (shown in green) and the standard MNI152 T1 1-mm^3^ brain template. The value of *z* in the horizontal plane represents the MNI *z*-coordinate. Abbreviation: AD, axial diffusivity; FA, fractional anisotropy; L, left; MNI, Montreal Neurological Institute; R, right.

**Figure 4:**
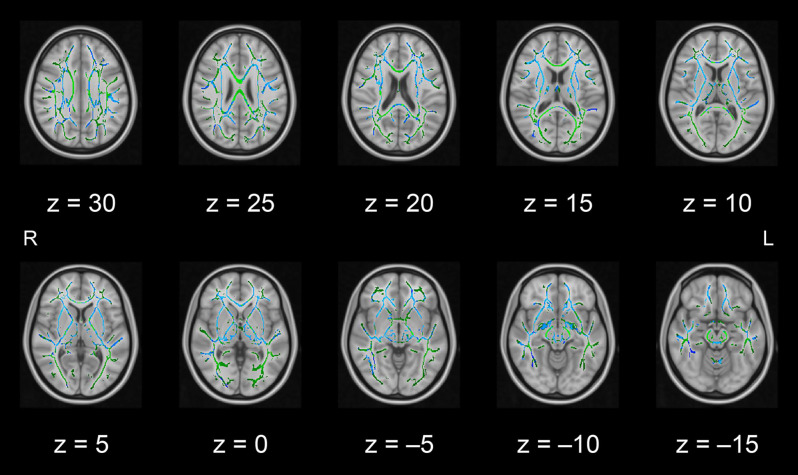
White matter fiber tracts showing significantly lower RD values in the intervention group than in the control group (displayed in blue). The results are overlaid on the mean FA skeleton (shown in green) and the standard MNI152 T1 1-mm^3^ brain template. The value of *z* in the horizontal plane represents the MNI *z*-coordinate. Abbreviation: FA, fractional anisotropy; L, left; MNI, Montreal Neurological Institute; R, right; RD, radial diffusivity.

In this pilot study, the diffusion metrics were compared between the intervention and control groups. Consistent with the hypothesized pattern, the intervention group showed in numerous white matter regions, including the left frontal area, significantly higher FA values or lower MD, AD, or RD values than the control group (see Figures 1–4, respectively). As shown in Figure 1; the bilateral anterior and superior corona radiata, external capsule, anterior and posterior limbs of the internal capsule, left genu and body of the corpus callosum, corticospinal tract, sagittal stratum (which includes the inferior longitudinal and inferior fronto-occipital fasciculi), retrolenticular part of the internal capsule, and fornix (cres)/stria terminalis had significantly higher FA values in the intervention group. As shown in Figures 2–4, these clusters overlapped with regions where significantly lower MD, AD, and RD values were observed in the intervention group. Furthermore, lower values of MD, AD, or RD were more widely found in regions such as the anterior, superior, and posterior corona radiata; external capsule; anterior and posterior limbs of the internal capsule; retrolenticular part of the internal capsule; genu, body, and splenium of the corpus callosum; corticospinal tract; sagittal stratum including the inferior longitudinal and inferior fronto-occipital fasciculi; superior and inferior longitudinal fasciculi; uncinate fasciculus; fornix; cingulum (cingulate gyrus); posterior thalamic radiation including the optic radiation; and midbrain regions. The findings from FA, MD, AD, and RD maps are detailed in Table 1. Importantly, the inverse pattern, i.e., lower values in FA or higher values in the other three metrics in the intervention group relative to the control group, was not found in any fibers.

**Table 1:** White matter structures showing significant differences in DTI metrics between the intervention and control groups.

**Labels of white matter structures included in clusters**	**MNI coordinates of the peak voxel**			**Number of voxels**
	***x***	***y***	***z***	
**FA (Intervention > Control)**				
Left ACR, SCR, EC, ALIC, PLIC, GCC, and BCC	−25	17	16	1,836
Right ACR, SCR, EC, and ALIC	26	13	18	1,081
Left EC	−31	11	−4	115
Right EC	35	−8	−11	20
Right ALIC and PLIC	21	−3	14	93
Left CT	−19	−22	−4	40
Left SS (includes ILF and IFOF) and RPIC	−40	−19	−10	193
Left fornix (cres)/ST	−31	−24	−7	7
**MD (Control > Intervention)**				
Bilateral ACR, SCR, PCR, EC, ALIC, PLIC, RPIC, GCC, BCC, SCC, CT, SS (includes ILF and IFOF), SLF, UF, fornix (cres)/ST, fornix (column and body of fornix), cingulum (CG), PTR (includes OR), and midbrain	45	0	−31	57,187
**AD (Control > Intervention)**				
Bilateral ACR, SCR, PCR, EC, ALIC, PLIC, RPIC, GCC, BCC, SCC, CT, SLF, fornix (column and body of fornix), PTR (includes OR), and midbrain, and right SS (includes ILF and IFOF), UF, fornix (cres)/ST, and cingulum (CG)	36	−36	22	34,288
**RD (Control > Intervention)**				
Bilateral ACR, SCR, PCR, EC, ALIC, PLIC, RPIC, GCC, BCC, SCC, CT, SS (includes ILF and IFOF), SLF, UF, fornix (cres)/ST, fornix (column and body of fornix), cingulum (CG), PTR (includes OR), and midbrain	30	6	−12	49,218
Right ILF	31	−86	−4	119
Right ILF	25	−88	−5	13

*Abbreviations: ACR, anterior corona radiata; AD, axial diffusivity; ALIC, anterior limb of the internal capsule; BCC, body of the corpus callosum; CG, cingulate gyrus; CT, corticospinal tract; DTI, diffusion tensor imaging; EC, external capsule; FA, fractional anisotropy; GCC, genu of the corpus callosum; IFOF, inferior fronto-occipital fasciculus; ILF, inferior longitudinal fasciculus; MD, mean diffusivity; MNI, Montreal Neurological Institute; OR, optic radiation; PCR, posterior corona radiata; PLIC, posterior limb of the internal capsule; PTR, posterior thalamic radiation; RD, radial diffusivity; RPIC, retrolenticular part of the internal capsule; SCC, splenium of the corpus callosum; SCR, superior corona radiata; SLF, superior longitudinal fasciculus; SS, sagittal stratum; ST, stria terminalis; UF, uncinate fasciculus*.

**Figure 5:**
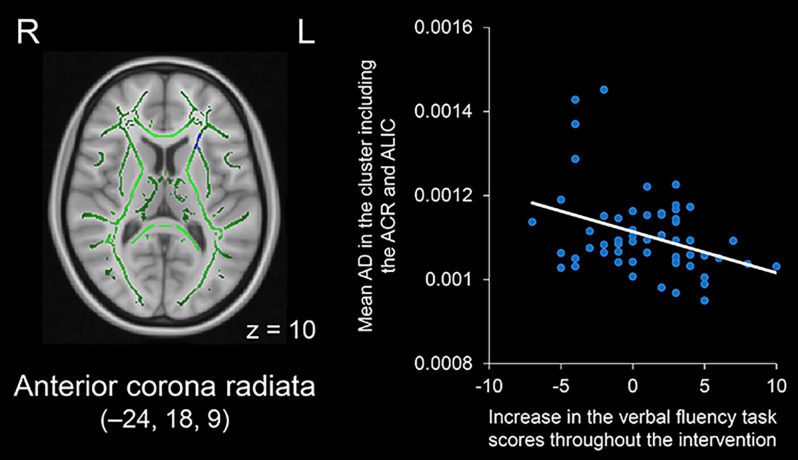
Negative correlation between increases in verbal fluency task scores throughout the intervention period and AD values in the cluster including the left ACR and ALIC (displayed in blue). The cluster is overlaid on the mean FA skeleton (shown in green) and the standard MNI152 T1 1-mm^3^ brain template. The value of *z* in the horizontal plane represents the MNI *z*-coordinate. MNI coordinates of the peak voxel are *x* = –24, *y* = 18, *z* = 9. Abbreviations: ACR, anterior corona radiata; AD, axial diffusivity; ALIC, anterior limb of the internal capsule; FA, fractional anisotropy; L, left; MNI, Montreal Neurological Institute; R, right.

As a complementary analysis, regression analysis was performed for each DTI index in relation to the score increases in the verbal fluency task throughout the intervention period. The results showed that a larger increase in task scores was associated with smaller AD values in clusters including the left anterior corona radiata, external capsule, or anterior limb of the internal capsule (see Figure 5). These clusters were located in regions where significant group differences in FA, MD, AD, and RD values were observed. The results of the regression analysis are summarized in Table 2. We found no significant correlations between increased task scores and other metrics, including FA, MD, and RD, in any fiber tract.

**Table 2:** White matter structures whose AD values showed negative correlations with increases in verbal fluency task scores throughout the intervention period.

**Labels of white matter structures included in clusters**	**MNI coordinates of the peak voxel**			**Number of voxels**
	***x***	***y***	***z***	
Left ACR and ALIC	−24	18	9	144
Left EC	−24	13	−11	32
Left EC	−23	20	−7	8

*Abbreviations: ACR, anterior corona radiata; AD, axial diffusivity; ALIC, anterior limb of the internal capsule; EC, external capsule; MNI, Montreal Neurological Institute*.

## Discussion

This pilot DTI study aimed to identify candidate structures for white matter alterations possibly induced by the PICMOR intervention program. To achieve this purpose, TBSS analysis was performed, and comprehensive DTI metrics including FA, MD, AD, and RD were compared between the intervention and control groups. We found significantly larger FA values or smaller values in the other three indices in the intervention group compared to the control group in numerous white matter fiber tracts, including the left anterior corona radiata, external capsule, and anterior limb of the internal capsule. By contrast, no region showed the inverse DTI index pattern. Furthermore, larger improvements in verbal fluency task scores from baseline to follow-up were associated with smaller AD values in several white matter regions, such as the left anterior corona radiata, external capsule, and anterior limb of the internal capsule. These findings suggest that conversation-based cognitive interventions have the potential to maintain or improve white matter fiber health in older adults and that left frontal white matter structures are candidate regions that contribute to the enhancement of verbal fluency by this intervention.

The DTI pattern identified in this study, i.e., higher FA values or lower MD, AD, or RD values in the intervention group than in the control group, is consistent with previous findings from cognitive intervention studies using comprehensive DTI metrics (Lovden et al., 2010; Engvig et al., 2012; Cao et al., 2016; de Lange et al., 2017). Given that normal aging is typically associated with decreases in FA or increases in MD, AD, or RD across numerous white matter tracts (Madden et al., 2009, 2012; Bennett and Madden, 2014), including the fiber tracts identified in the present study, the DTI pattern at follow-up can be interpreted as evidence that age-related alterations in the white matter microstructure were attenuated by conversation-based cognitive interventions. Although the neurobiological interpretability of these DTI patterns is limited (Beaulieu, 2014), comprehensive use of DTI metrics would still be useful for characterizing intervention/training-induced changes in the white matter microstructure. This pilot study demonstrated the applicability of comprehensive DTI analyses in intervention studies and extended previous findings by demonstrating that intervention methodologies based on daily social activities like conversations have the potential to improve or maintain white matter fiber health in older adults. Considering the difference in structured conversations for the intervention group enabled by a chair robot and unstructured free conversations for the control group, the structured nature of conversations may play an important role in the possible intervention effect on white matter microstructure. As noted earlier, structured conversations offer opportunities to train executive functions, such as flexibility, planning, working memory, and response inhibition. Indeed, verbal fluency ability was improved by structured conversations (Otake-Matsuura et al., 2021). Together with the behavioral findings, the PICMOR method could have a beneficial effect on older adults’ health at both the behavioral and neural levels.

In the present study, a larger increase in verbal fluency task scores throughout the intervention period was associated with a smaller AD value in the left anterior corona radiata, external capsule, and anterior limb of the internal capsule (see Figure 5). These regions showed higher FA and lower MD, AD, and RD in the intervention group than in the control group (see Figures 1–4, respectively). The present findings are consistent with neuropsychological evidence demonstrating the contribution of the left frontal region to verbal fluency (Stuss et al., 1998; Baldo et al., 2006, 2010; Robinson et al., 2012; Biesbroek et al., 2016, 2021; Chouiter et al., 2016; Li et al., 2017; Thye et al., 2021). For instance, voxel-based lesion-symptom mapping studies have revealed that damage to white matter tracts located in the left hemisphere, such as the anterior corona radiata, external capsule, or anterior limb of the internal capsule, is associated with deficits in verbal fluency (Chouiter et al., 2016; Thye et al., 2021). Likewise, DTI studies have shown that white matter properties in the left frontal region are associated with verbal fluency (Theilmann et al., 2013; Rodriguez-Aranda et al., 2016). For example, one DTI study examined patients with Parkinson’s disease and reported that verbal fluency scores were correlated with FA values in several regions, including the left anterior corona radiata (Theilmann et al., 2013). Taken together, the present findings suggest that left frontal white matter structures, including the anterior corona radiata, external capsule, and anterior limb of the internal capsule, are candidates for the neural underpinnings responsible for the beneficial effects of PICMOR on verbal fluency in older adults. Interestingly, our preliminary study using resting-state functional MRI revealed that left frontal cortical regions located near the regions identified in the present DTI study showed differential functional connectivity in the two groups (Sugimoto et al., 2020). Compared to the control group, the right prefrontal cortex and temporal pole in the intervention group had increased resting-state functional connectivity with the left prefrontal cortex, which is the most important cortical region for verbal fluency (Costafreda et al., 2006; Wagner et al., 2014). The strength of functional connectivity between the left prefrontal cortex and the right temporal pole was positively correlated with increased verbal fluency task scores, whereas the strength between the left and right prefrontal cortices showed no significant correlation with the task scores (Sugimoto et al., 2020). Further analysis using voxel-based morphometry demonstrated that the right prefrontal region had increased volumes in the intervention group compared to the control group, although the volume of this region was not correlated with task scores (Sugimoto and Otake-Matsuura, 2022). In the present DTI study, higher FA and lower MD, AD, and RD in the intervention group were observed in both left and right frontal regions, although a significant correlation with task scores was identified only on the left side. Thus, these findings could be interpreted as evidence that conversation-based cognitive interventions have the potential to induce structural and functional changes not only on the left side of the frontal area as a core region for verbal fluency but also on the right side of this area as a supplementary region that indirectly supports verbal fluency. This idea should be further examined in future studies by employing equivalent criteria for statistical significance among different modalities.

Our findings are limited by the lack of comparable MRI data at baseline. Any difference observed in the post-intervention period cannot be fully considered an intervention effect because such difference may have already existed before the intervention. Although we adopted the random allocation of participants to the study groups (Otake-Matsuura et al., 2021) and found no significant group differences at baseline in the task scores of almost all cognitive tests, including the verbal fluency task (Sugimoto and Otake-Matsuura, 2022), we cannot rule out the possibility of baseline differences. To examine whether the possible white matter alterations identified in this pilot study were indeed induced by PICMOR-based conversations, it is necessary to collect longitudinal DTI data including both pre- and post-intervention periods and to compare longitudinal changes between intervention and control groups. Despite this limitation, we successfully identified the hypothesized DTI pattern, i.e., intervention-induced increases in FA or decreases in MD, AD, or RD, which should be confirmed by future research using a longitudinal study design.

## Conclusion

Although we cannot conclusively identify the neural underpinnings of the beneficial effects of PICMOR on cognitive function due to the lack of DTI data at baseline, this pilot study successfully identified candidate structures by comparing comprehensive DTI metrics at follow-up between the intervention and control groups. The observed DTI pattern is consistent with those identified in other cognitive intervention studies. Taken together with ample evidence from aging research that normal aging is associated with changes in DTI indices, the present findings suggest that conversation-based cognitive interventions have the potential to maintain or improve white matter fiber health in adults affected by age-related alterations. Furthermore, the findings from the regression analysis suggest that left frontal white matter structures are candidates that contribute to the intervention-induced enhancement of verbal fluency. Definitive conclusions may be obtained by comparing in future research longitudinal changes in DTI measures from baseline to follow-up in candidate regions.

## Data Availability Statement

The datasets presented in this article are not readily available because joint research agreement is required for data sharing. Requests to access the datasets should be directed to Hikaru Sugimoto, hikaru.sugimoto@riken.jp.

## Ethics Statement

This study was reviewed and approved by the Institutional Review Board of RIKEN. The participants provided their written informed consent to participate in this study.

## Author Contributions

MO-M designed the study. HS collected and analyzed the data and wrote the manuscript under the supervision of MO-M. All authors contributed to the article and approved the submitted version.

## Conflict of Interest

The authors declare that the research was conducted in the absence of any commercial or financial relationships that could be construed as a potential conflict of interest.

## Publisher’s Note

All claims expressed in this article are solely those of the authors and do not necessarily represent those of their affiliated organizations, or those of the publisher, the editors and the reviewers. Any product that may be evaluated in this article, or claim that may be made by its manufacturer, is not guaranteed or endorsed by the publisher.

## Footnotes

^1^ https://fsl.fmrib.ox.ac.uk/fsl/fslwiki

^2^ http://www.lin4neuro.net/lin4neuro/

^3^ https://www.mrtrix.org/

## Abbreviations

AD: axial diffusivity
DTI: diffusion tensor imaging
FA: fractional anisotropy
FDT: FMRIB’s Diffusion Toolbox
FMRIB: Functional MRI of the Brain
FSL: FMRIB’s Software Library
ICBM: International Consortium of Brain Mapping
JHU: Johns Hopkins University
MD: mean diffusivity
MMSE-J: Japanese version of the Mini-Mental State Examination
MNI: Montreal Neurological Institute
MRI: magnetic resonance imaging
PICMOR: Photo-Integrated Conversation Moderated by Robots
RCT: randomized controlled trial
RD: radial diffusivity
SD: standard deviation
TBSS: tract-based spatial statistics
TIV: total intracranial volume.
